# Health Tract No. 326

**Published:** 1868-07

**Authors:** 


HEALTH TRACT No. 326.

### TRYING TO GET SICK.

Medical Philosophers assure us that three-fourths of ordinary diseases are avoidable, could be prevented if we acted up to the knowledge we have but some are not only careless of their health, make no effort to avoid disease but actually seem determined to get sick; the reader has done this many a time, and will continue to do it in too many cases. You have often come to the table, glanced over it, saw nothing which invited your appetite and got as mad as a hornet in a minute. At yourself? Oh no, but at your wife, or cook, because something was not on the table which you could relish. But you could not yourself tell what you wanted, and acted as if you expected them to dive deep into your gluttonous maw and divine what would please you; mayhap you wanted forty different dishes on the table that you might make your sovreign selection, leaving the three dozen others to be thrown away and then the next thing would be a virtuous indignation at the waste in the house, and the expense of living. Why half the men don't deserve wives, and yet they are the very ones who get the best. Out upon it you unreasonable, ignorant churls! The real state of the case was, you were not hungry; you had not taken exercise enough since the last meal to cause the appropriation of all the nutriment in the system; until that is done, nature prepares none of the fluid which causes the feeling of hunger; your wife could not help that, nor your cook, you might have helped it yourself, had you not been too lazy to work yourself into an appetite; but determined to make yourself sick, you sat down very grumpily, with both elbows on the table, or beating a tattoo, both indicating passion, presently you began to nibble at this and nibble at that, and before you knew it, had eaten a hearty meal; but having crowded into the system what nature did not need, she refused to appropriate it, and you began to "feel bad all over," the system was opppressed, you were cross, peevish, fretful, complaining, and finally was more or less ailing, if not actually sick for several days. Even a pig will not eat when he is not hungry, but you, a bigger pig, do.

